# Association of Seedling Vigour and Salinity Tolerance in Field Pea

**DOI:** 10.3390/plants13223140

**Published:** 2024-11-08

**Authors:** Babu R. Pandey, Ramesh R. Puri, Arun S. K. Shunmugam, Dianne Noy, Garry M. Rosewarne

**Affiliations:** Agriculture Victoria, Grains Innovation Park, 110 Natimuk Road, Horsham, VIC 3400, Australia; rameshrajpuri@gmail.com (R.R.P.); garry.rosewarne@agriculture.vic.gov.au (G.M.R.)

**Keywords:** seedling vigour, salinity tolerance, dry weight reduction, Na^+^, K^+^

## Abstract

Soil salinity results in reduced productivity in field peas, making soil salinity tolerance a critical breeding objective. In this study, four pot experiments were carried out in semi-controlled environments over four consecutive years to assess the contribution of seedling vigour to salinity tolerance at the seedling stage. Split-plot designs were used to assess the effect of salt stress (sodium chloride solution at 16 dSm^−1^) and control conditions. Extensive sets of advanced breeding lines were used in 2018–2020 to assess growth differences in relation to the treatment, with elemental analysis used on a subset of 15 lines in 2021. A salt tolerance index (STI) was defined as a proportion of shoot biomass under salt stress (DWstress) relative to the shoot dry weight under control (DWctrl). Visual scores of salt stress were recorded on a 1–10 scale (1 = tolerant, 10 = susceptible) from salt stress treatments. The consistent positive and significant correlations (*p* < 0.01) between shoot DWctrl and DWstress indicated that vigorous genotypes maintained higher shoot DWstress. Both the shoot DWctrl and shoot DWstress had negative and significant (*p* < 0.01) correlations with visual scores of salt stress. Shoot DWstress showed strong positive correlations with STI (*p* < 0.01). Both the shoot DWctrl and Shoot DWstress had negative correlations (*p* < 0.01) with shoot Na^+^ whereas shoot DWstress had a positive correlation (*p* < 0.05) with root Na^+^ concentration. The results indicated that seedling vigour (measured as shoot DWctrl) contributed to salinity tolerance by maintaining improved shoot DWstress, limiting Na^+^ deposition in shoot and enduring less tissue damage in field pea seedlings. Additional field evaluations are required to establish the correlations of tolerance at seedling stage with yield under saline conditions. The insights obtained from this study may assist field pea breeders in identifying salt-tolerant parent plants, offspring, and breeding lines during the initial growth phases.

## 1. Introduction

Soil salinization is a significant abiotic stress factor that has a global impact on crop yields. Approximately 6% of the Earth’s total land area, including 20% of arable land and 33% of irrigated land, is under the threat of salinity [1,2,3]. The expansion of salt-affected areas worldwide is expected to occur at a rate of about 10% per year, primarily due to factors such as low precipitation, high surface evaporation, rock erosion, irrigation with saline water, and inadequate farming practices [4]. In Australia, soil salinity poses a significant challenge to crop production in many regions of southern Australia. Particularly during periods of low rainfall, high salinity is a contributing factor to crop losses or reduced yields.

Pulses have a recognized susceptibility to soil salinity [5]. Soil salinity as low as 1.5 dS/m is detrimental to pea yield [6]. Saline soils in cropping regions in Australia has salinity ranging from 4 to 16 dS/m [7]. The presence of salinity stress has a substantial negative impact on plant growth and can lead to a significant decrease in crop yield [8]. Initially, soil salinity is known to suppress plant growth in the form of osmotic stress which is then followed by ion toxicity [9,10,11]. High salt accumulation in soils reduces the water absorption capacity of root systems, resulting in an osmotic stress and water loss in leaves [12]. High Na^+^ inhibits plant uptake of other cationic elements, such as K^+^, Na^+^, and K^+^, and homeostasis plays a vital role in the growth and development of higher plants under salt conditions due to K^+^–Na^+^ interaction. High Na^+^ can be associated with K^+^ deficiency [13].

Salt stress impacts multiple facets of plant growth, including photosynthesis, biomass production, yield, and the production of metabolites in leaf tissues [14,15]. Salt tolerance is a complex phenomenon, and plants exhibit diverse adaptations at the subcellular, cellular, and organ levels. These adaptations include regulating stomatal function, maintaining ion balance, balancing hormonal levels, activating antioxidant defence mechanisms, osmotic adjustment, and preserving tissue water status. These adaptations are crucial for the successful growth of plants in saline environments [16].

The mechanisms underlying plant salinity tolerance can be categorized into three types: (1) osmotic tolerance, which involves the recognition and signalling processes that occur prior to the accumulation of sodium (Na^+^) in the shoot, leading to a decrease in growth rate; (2) ion exclusion, where the roots sequester ions to limit their accumulation in the shoot; and (3) tissue tolerance, where high concentrations of Na^+^ in the shoot are compartmentalized within vacuoles to minimize their harmful effects [2,17,18]. Shahid et al. [19] reported that tolerant field pea genotypes maintained favourable K^+^/Na^+^ and Ca^2+^/Na^+^ ratios by limiting the transport of Na^+^ into the shoot. This observation suggests that these ratios could serve as reliable indicators of the plant’s response to salt stress. Balal et al. [20] also observed similar results in salinized citrus root stocks.

Multiple approaches can be applied to minimize the effects of salinity. Utilization of genetic variation in available germplasm is one the best approach because it provides opportunity to select genotypes to grow under saline conditions and/or to use them as parents in a breeding program [21]. It is widely recognized that varietal variances offer substantial genetic diversity for enhancing crop development [22]. The screening of crops for tolerance enables breeding programs to identify of genotypes that exhibit both high salt tolerance and promising yield potential.

In recent years, the significance of salinity tolerance in field pea has been progressively emphasized in Australia owing to a geographical change of crop production towards the environments characterized by shorter seasons, reduced water availability, and marginal soils with higher transient soil salinity [23]. Growth maintenance, defined by an increase in plant biomass, has been broadly accepted to be a good indicator of salinity tolerance in field peas [19,23], tomato [2]), and wheat [15]. We conducted four pot experiments to study the contribution of vigour in salinity tolerance of field pea seedlings in 2018–2021. In 2021, we selected fifteen field pea genotypes from the 2020 experiment to study the association of Na^+^, K^+^, and their ratios for salinity tolerance.

## 2. Materials and Methods

### 2.1. Plant Material and Experimental Design

Three sets of advanced field pea breeding lines and check varieties were screened for salinity tolerance in 2018, 2019, and 2020 (Supplementary Tables S1, S2 and S3, respectively). There were 146 (2018 and 2019) and 147 (2020) field pea breeding lines and check varieties sown in an igloo at Grains Innovation Park, Horsham, Victoria. Fifteen genotypes with a wide range of salinity tolerance were selected from the 2020 experiment. The fourth experiment was designed with the selected fifteen field pea genotypes (Supplementary Table S4) in 2021 to study the inherent mechanism for salinity tolerance.

The experiments were laid out in a split plot design with two replicates in 2018–2020 and with three replicates in 2021. Salinity treatments (treatment and control) were the main-plot factors, and the genotypes were sub-plot factors. The experiments were sown in tapered cylindrical pots (14 cm height, 13 cm top diameter, and 11 cm bottom diameter). The pots were filled to within approximately 3.0 cm of the top with a potting mix of a 1:1 ratio of coarse river sand and plumbers grit (blue stone chips). The pots were initially watered with a heavy stream of water to rinse any impurities from the potting medium. Then six seeds per pot were sown, one in the centre and five around it, at approximately a 2 cm depth. Pots were watered lightly each day until they emerged. Hydroponic nutrient solution (Manutec Pty. Ltd., Cavan, South Australia) was applied in both the control and salt-treated pots during each salt stress treatment.

### 2.2. Salinity Applications

Salt treatment was applied by flooding the pots with sodium chloride (NaCl) solution after ten days of emergence. Salt solution was prepared using NaCl dissolved stock solution as described by [23]. Electrical conductivity of the prepared solution was measured with an EC meter. According to empirical formula used by [24], 1 deci siemens per metre (dS/m) equals to 0.07% concentration under standard temperature (25 °C). The ten-day-old seedlings were flooded with a starting concentration of 4 dS/m (=0.28% or 2.8 g/dm^3^) for four days. On the fifth day, the concentration was increased to 8 dS/m. The salt concentration was increased by 4 dS/m every four days and increased up to 16 dS/m (=1.12% or 11.2 g/dm^3^). The final concentration of NaCl solution was applied for 16 days. Altogether, the salt treatments were applied for four weeks. The control treatment was applied by flooding the pots with normal water.

### 2.3. Measurements

Shoot biomass was harvested from all the pots and was oven-dried at 70 °C for 72 h to record the dry weights. The salinity tolerance score and screening method were based on a visual growth response scale (1–10), established for lentil [25] and adapted for field pea as described in Table 1. From the 2021 experiment, the roots were also harvested, washed with distilled water, and oven-dried at 70 °C for 72 h to record dry weight. Salt tolerance index (STI) was estimated as proportion of shoot dry weight under salt stress relative to shoot dry weight under control as:

$$STI=\frac{DWstress}{DWctrl},$$

where DW_ctrl_ is dry weight of control and DW_stress_ is dry weight of salt treated plants.

### 2.4. Determination of Tissue Elements

The shoot and root biomass samples from the 2021 experiment were subjected to tissue elemental analysis. A representative sub-sample of dried plant tissue was weighed into a Pyrex tube and digested by a mixture of nitric and perchloric acids in a heated aluminium block [26]. After appropriate dilution, the concentrations of analytes in the digestate were determined by Inductively coupled plasma—optical emission spectrometry (ICP-OES) (Agilent 5100 DV) at Agilent Technologies Australia Pty Ltd. 679 Springvale Rd. Mulgrave VIC 3170, Australia. The tissue analysis included estimation of Na^+^, K^+^, and Ca^2+^ concentrations in the shoot and root. K^+^/Na^+^ and Ca^2+^/Na^+^ were derived for both shoot and root.

### 2.5. Statistical Analysis of Data

Data were analysed using the GenStat 21st Edition Statistical Software [27]. A linear mixed model approach was used to examine the effects of salinity treatment, genotypes, and their interactions on biomass production of field pea seedlings. Salinity treatment and the genotypes were fitted as fixed effect while the block effects were fitted as random. As the number of plants emerged per pot ranged from 2 to 6, it was fitted as a fixed effect in the model. Predicted means were used to study relationships between the variables. Correlation analysis was conducted for all the traits measured in 2021 using the cor and corrplot functions from the corrplot package in R [28].

The principal component analysis (PCA) was used to describe the dataset from the 2021 experiment. Based on their usefulness to predict the salinity tolerance, 11 variables were selected to use in the PCA. The PCA analysis was carried out on standardized data using the prcomp function in R [29] and a biplot was drawn using factoextra R package [30].

## 3. Results

### 3.1. Effects of Salinity on Shoot Biomass

**Figure 1:**
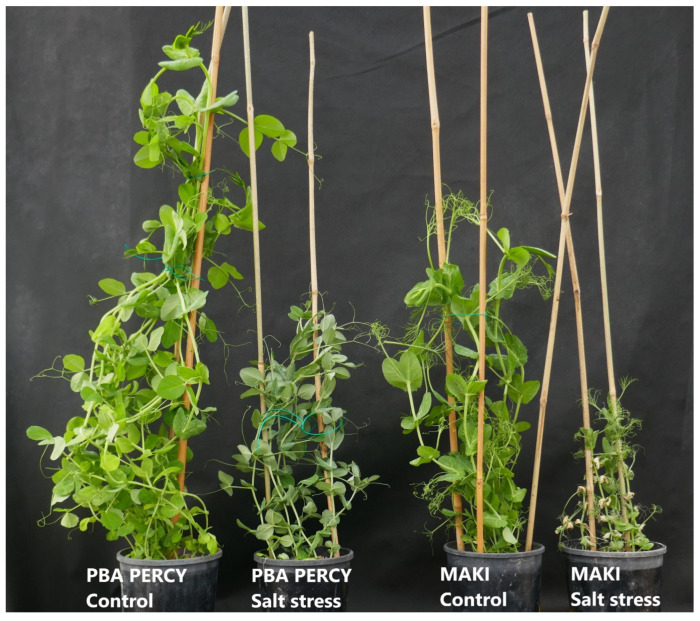
Typical differences in plant growth among selected check varieties under control and salt stress. Plants were grown under control and salt stress for four weeks. The pictures were taken before the harvest in 2021. Maki is a susceptible and PBA Percy is moderately tolerant check variety.

There was a significant (*p* < 0.001) reduction in shoot biomass due to salt treatment in all the experiments (2018–2021) (Table 2). Likewise, number of plants per pot used as a covariate was also significant (*p* < 0.001) in all the experiments. Figure 1 presents the photos of selected check varieties under control and salt treatments.

### 3.2. Correlations Between Dry Weights, STI and Visual Scores

**Figure 2:**
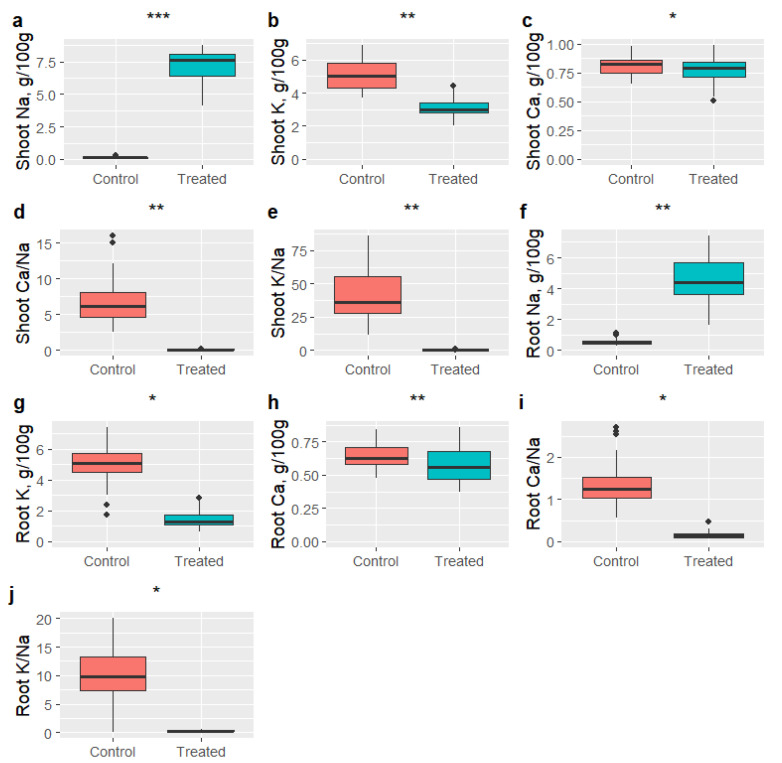
Effect of salt treatment on elemental concentrations in shoot and root in 2021. Contents of elements in shoot (**a**–**c**) and roots (**f**–**h**), ratios of elements in shoot (**d**,**e**), ratios of elements in root (**i**,**j**). The bottom, middle and top line of the box plot represent the first, second and the quartiles of the data, respectively. Differences between the treatments were tested with analysis of variance (ANOVA). ***, **, and * indicate that the means were significantly different at 0.001, 0.01, and 0.05 level of significance, respectively.

There were positive and significant correlations between shoot DWctrl and shoot DWstress in all the experiments (Table 3). The correlations were consistent across the experiments (r = 0.46 in 2018, 0.59 in 2019, 0.47 in 2020, and 0.94 in 2021). Similarly, there were negative and significant correlations between DWstress and STI (r= −0.78, −0.78, −0.76, and −0.80 in 2018, 2019, 2020, and 2021, respectively). Further, visual score of salt stress had significant and negative correlations with DWstress (r = −0.78, −0.69, −0.67, and −0.89, in 2018, 2019, 2020, and 2021, respectively), and significant positive correlations with STI (r = 0.56, 0.66, 0.47, and 0.76, in 2018, 2019, 2020, and 2021, respectively) (Figure 2). Shoot DWctrl showed inconsistent correlations with STI. The correlations were positive significant in 2018 and 2020 (r = 0.19 and 0.18, respectively), negative significant in 2021 (r = −0.55) and non-significant in 2019. However, DWctrl showed consistent negative correlations with visual scores of salt stress. The correlations ranged from −0.18 in 2020 to −0.82 in 2021.

### 3.3. Effect of Salt Stress on the Elemental Contents in Shoot and Root in 2021

#### 3.3.1. General Effects of Salinity on Elemental Concentration

Salt treatment significantly increased Na^+^ concentration in shoot (control: 0.15 ± 0.012 g/100 g vs. salt stress: 7.08 ± 0.05 g/100 g) and root (0.55 ± 0.03 vs. 4.61 ± 0.25), and significantly reduced Ca^2+^ concentrations (g/100 g) in shoot (0.81 ± 0.006 vs. 0.77 ± 0.01) and root (0.65 ± 0.03 vs. 0.58 ± 0.05) (Figure 2). Similarly, K^+^ concentration (g/100 g) was also significantly reduced in shoot (5.08 ± 0.31 vs. 3.09 ± 0.18) and root (5.08 ± 0.30 vs. 1.47 ± 0.07). Since salt treatment increased the shoot and root Na^+^ concentrations and reduced the K^+^ and Ca^2+^ concentrations which resulted in decrease in K^+^/Na^+^ and Ca^2+^/Na^+^ ratios in salt stress.

#### 3.3.2. Genotypic Differences in Elemental Concentrations Under Salt Stress

The fifteen genotypes were grouped into two groups—‘tolerant’ and ‘sensitive’ based on their STI. The six genotypes which had 0.40 or less STI were grouped as tolerant and the remaining nine genotypes, which had >0.40 STI, were grouped as sensitive (Supplementary Table S4).

**Figure 3:**
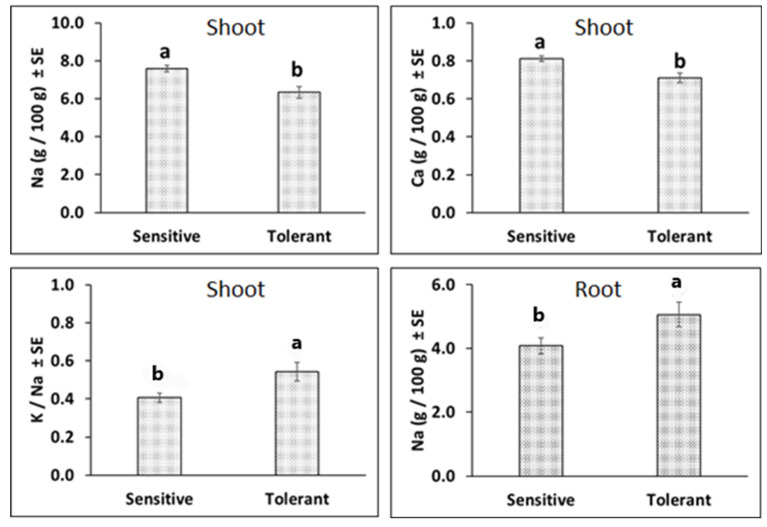
Effect of salt stress on elemental concentration of tolerant and susceptible genotypes in field pea shoot and root. Values are mean values ± standard error of mean (SE). The means of the tolerant and susceptible groups were tested using a *t*-test. The same alphabet above the bars are not statistically significant at 0.05 level of significance. n = 27 and 18 for the susceptible and tolerant group, respectively.

Under salt stress, the tolerant group of genotypes had significantly lower concentrations of Na^+^ and Ca^2+^ than sensitive group of genotypes in shoot (Figure 3). Further, the tolerant group had significantly higher K^+^/Na^+^ ratio than that of the sensitive group in shoot. Similarly, the tolerant groups had a significantly higher Na^+^ concentration than the sensitive groups in roots. The differences for K^+^ and Ca^2+^/Na^+^ in shoot, and K^+^, Ca^2+^, K^+^/Na^+^, and Ca^2+^/Na^+^ in root were not significantly different between the tolerant and sensitive groups.

### 3.4. Correlations of Dry Weights, STI and Visual Scores with Elemental Concentrations and Their Ratios

Table 4 presents correlations between dry weights, STI, and visual scores of salt stress with elemental concentrations and their ratios in shoot and root. The visual scores, elemental concentrations, and their ratios are presented only for salt stress treatment. Shoot DWctrl had negative significant correlation with shoot Na^+^ and positive significant correlation with root K^+^ (Table 4). Shoot DWstress had negative significant correlations with shoot Na^+^ and root Ca^2+^/Na^+^; but positive significant correlations with root Na^+^, root K^+^, and shoot K^+^/Na^+^. STI had negative significant correlations with shoot Na^+^, root K^+^/Na^+^, and root Ca^2+^/Na^+^ but positive significant correlations with shoot K^+^, root Na^+^, shoot K^+^/Na^+^, and shoot Ca^2+^/Na^+^. Visual scores of salt stress showed similar correlations with the elemental concentrations and their ratios. The correlations of visual scores were positive significant with shoot Na^+^ and root Ca^2+^/Na^+^, negative significant with shoot K^+^, root Na^+^, root K^+^, and shoot K^+^/Na^+^.

### 3.5. Principal Component Analysis

A principal component analysis (PCA) was conducted to reduce complexity in the data and explore relationships among the traits measured in 2021. In this study, the first three principal components (PC) had eigen values >1 (Supplemental Table S5) indicating that the variance explained by the PCs exceeded that explained by any single original variable in the dataset. This criterion was utilized as a threshold to determine the number of principal components to retain.

**Figure 4:**
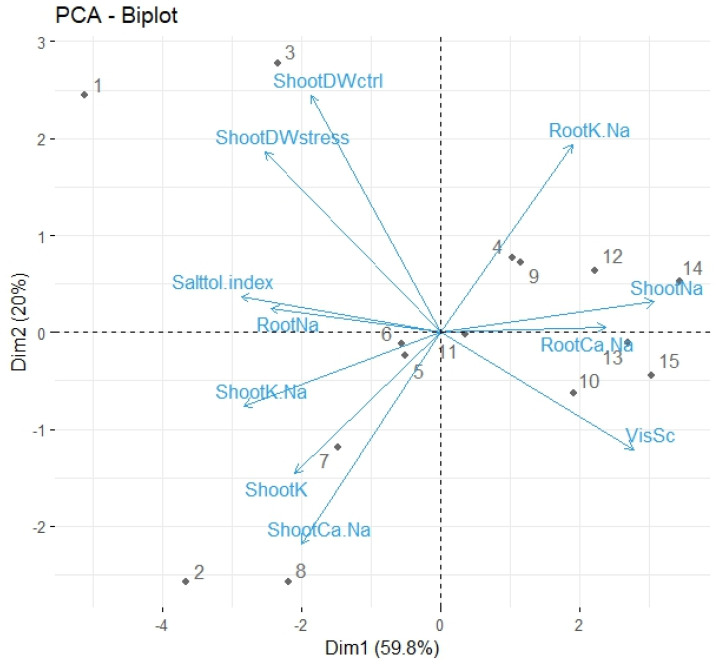
Biplot of 15 field pea genotypes, based on the variance in 11 traits, explained by two PC axes (Dim 1 and Dim 2). The numbers 1 to 15 indicate genotype ranks in ascending order of salt tolerance index.

PC1 explained 59.8% of the total variability in the data. The highest contributing trait for PC1 was the shoot Na^+^ concentration under salt stress, followed by STI, shoot K^+^/Na^+^, and visual scores of salt stress (Table 5). The analysis suggested that the PC1 represented traits related to susceptibility to salt stress. The PC1 clearly separated the two groups of genotypes (tolerant and sensitive) on the axis. In the biplot, the genotypes are presented as numbers 1–15, representing their rankings in ascending order of STI (Figure 4). The field pea genotypes at the lower end (1–8, except 4) of PC1 were able to retain high shoot dry weight and low shoot Na^+^ concentration under salt treatment. The genotypes at the higher end (9–15) were the ones which had higher STI, had higher shoot Na^+^ under salt stress and had higher visual scores of salt stress. The genotypes 1 (OZP0812) and 3 (PBA Percy) formed a distinct group in quadrant 2 due to their high shoot DWctrl and shoot DWstress. The genotype 4 fell on the sensitive side despite having lower STI. This could be because of its relatively higher visual score for salt stress (6.36) and higher shoot Na^+^ (7.80 g/100 g) concentration.

PC2 accounted for an additional 20% of the total variability in the data. Traits related to plant growth (shoot DWctrl and shoot DWstress), shoot Ca^2+^/Na^+^ under salt stress, root K^+^/Na^+^ under salt stress and shoot K^+^ concentration under salt stress had major contribution to the PC2. The analysis suggested that PC2 represented a combination of traits related to biomass and concentrations of elements required to salt tolerance in the shoot.

PC3 explained 12.5% of variance in the data related to salinity tolerance traits. Root Ca^2+^/Na^+^ under salt treatment was the highest contributing traits to PC3 followed by root Na^+^ and shoot K^+^ under salt treatment (Table 4).

## 4. Discussion

The global agricultural sector is facing a growing challenge in the form of soil salinity, which adversely impacts crop production [31]. To develop crops with enhanced tolerance to salinity, it is necessary to employ a screening technique that is both rapid and dependable. Conducting salt screening experiments in field is challenging due to variations in soil composition and environmental factors. Furthermore, creating consistent and controlled levels of salinity proves to be a challenging task, making it difficult to achieve reproducible screening outcomes in field settings. Identification of key plant traits related to salinity tolerance and understanding the underlying process of tolerance have a great potential to aid breeding efforts in developing tolerant cultivars. We studied contribution of seedling vigour and underlying process of salinity tolerance in field pea seedlings by conducting four pot experiments in a semi-controlled environment.

In our study, shoot DWctrl (seedling vigour) had moderate but consistent associations with enhanced shoot DWstress and lower tissue damage measured as visual score of salt stress (Table 3). Similarly, shoot DWstress had moderate but consistent associations with lower STI. The biplot from the PCA also clustered the traits related to seedling vigour (shoot DWctrl and shoot DWstress) on the same side of PC1 along with the other traits related to salinity tolerance (root Na^+^, shoot K^+^/Na^+^, shoot K^+^, shoot Ca^2+^/Na^+^, and salt tolerance index) and salt tolerant genotypes (Figure 4). The results indicated that although seedling vigour did not show consistent correlations with STI, it contributed to salinity tolerance of the field pea seedlings by maintaining biomass under salt stress and reducing tissue damage. This was also further supported by results from elemental analysis in 2021 where seedling vigour was associated with lower Na^+^ in shoot and higher root K^+^ under salt stress (Table 4). Several studies have shown that the traits related to plant growth are a good indicator of salinity tolerance. Quamruzzaman et al. [32] observed in glasshouse experiments that the traits related to plant vigour such as plant height and biological yield were highly associated with grain yield of wheat under saline conditions. Akram et al. [33] and Carpici et al. [34] reported that seedling biomass was correlated with salinity tolerance in maize. Pailles et al. [2] investigated a diverse range of traits related to salinity tolerance in tomato. They concluded that fresh weight of the seedlings was one of major traits related to salinity tolerance. Dong et al. [35] also observed that the fresh weight of cowpea seedlings under control and saline conditions were moderately correlated (r = 0.57) in glasshouse conditions indicating that seedling vigour was associated with salinity tolerance. Similar results of association of seedling vigour with salinity tolerance were reported in canola [36], alfalfa [37], mung bean [38].

There were similar results reported from studies in field pea as well. Shahid et al. [19] screened 30 field pea genotypes for salinity tolerance at seedling stage. They observed that traits related to seedling vigour such as shoot length, root length, fresh weight, and dry weights of the seedlings were positively correlated with K^+^/Na^+^. Leonforte et al. [23] studied a collection of field pea germplasm for salinity tolerance under similar conditions as in our study. Most of the germplasm with salinity tolerance had traits such as long internodes and profuse basal branching which supported higher biomass. Some field studies have reported similar relationships between vigour and yield. For example, shoot biomass was associated with yield of chickpea under saline conditions in field [39].

Measurements of biomass or grain yield under control could be used as a selection criterion for salinity tolerance. These parameters integrate the response of the physiological processes to salinity at the whole plant level [40,41,42,43]. Richards [44] argued that improving vigour and yield potential under non-saline conditions would help the yield of cereal crops under saline conditions. In an assessment of a set of barley nested association mapping population, Saade et al. [45] observed that high yielding varieties under control conditions also yielded high under saline conditions in field. El-Hendawy [46] compared two wheat varieties (one susceptible and one moderately tolerant to salinity) under field saline conditions and found that the moderately tolerant variety yielded higher than the susceptible variety under both controlled and saline conditions. We reported results from studies based on seedling stage of the plant. Further evaluations are required to establish correlations of seedling tolerance with that of adult plants under field saline conditions.

We studied elemental concentrations of Na^+^, K^+^, and Ca^2+^ in shoot and root in 2021-experiment. In Pearson’s correlation analysis, salinity tolerance (indicated by higher STI) was associated with lower shoot Na^+^ but higher root Na^+^ concentration, shoot K^+^/Na^+^, and shoot Ca^2+^/Na^+^ under salt stress (Table 4). The genotypes were grouped into two groups based on STI (Supplementary Table S4). Under salt stress, the tolerant genotypes (>0.60 STI) had significantly lower shoot Na^+^ concentration but significantly higher root Na^+^ concentrations and shoot K^+^/Na^+^ than susceptible (<0.60 STI) genotypes, respectively (Figure 3). The results indicated that tolerant genotypes excluded toxic Na^+^ from shoot tissues by depositing them on root. The exclusion of toxic ions is one of the common mechanisms of adaptation to salinity in plants. Maintenance of homeostasis is important to effectively tolerate salinity. In saline environment, Na^+^ and K^+^ are highly competitive for uptake and transport in the plant because of their similar physio-chemical properties [47] resulting in the disruption of essential cellular metabolism [3,48,49,50]. Therefore, salinity tolerant genotypes demonstrate affinity to uptake K^+^ over Na^+^ and to limit up-taken Na^+^ ions in root tissues. Several research works have reported exclusion mechanism involved in adaptation of crops to salinity, for example soybean [51], alfalfa [52], and wheat [46].

Results from our study contradicted with that of Temme [53] who reported the vigorous genotypes at control were less tolerant to salinity. This might be due to the different mechanisms of salinity tolerance in field peas (exclusion of Na^+^) than in the sunflower (vacuolar sequestration of Na^+^). Plant species demonstrate a wide variation in salt tolerance mechanism. The tolerance mechanism might vary even within species [54]. Identification of physiological or biochemical indicators for each species is desirable for application in a selection program [55]. From similar study in field pea seedlings, Leonforte et al. [23] also observed that the salt tolerant lines had lower Na^+^ concentrations in shoot, indicating that Na^+^ exclusion was the mechanism of salt tolerance.

In our study, the genotypes with improved seedling vigour under control maintained higher biomass and lower shoot Na^+^ under stress. Higher biomass may be useful in diluting Na^+^ and delay its toxicity effects in plants [56]. Long internodes (*le* gene) provide most of the vigour in field pea, but modern varieties are mostly semi-dwarf with shorter internodes. Within semi-dwarf plant type, there is a scope to increase vigour by making selection for larger plant structure and relatively longer internodes [23]. The vigour before anthesis has been shown to be important for yield under low yielding environments in different crops for example, in wheat [57], in *Brassica juncea* canola [58], and in field pea [59]. With their improved canopy cover, vigorous crops at early growth stage reduce soil evaporation and increase water use efficiency [60,61].

The selection of seedling vigour/early vigour has a potential to improve not only salt tolerance but also yield under low yielding environments. Selecting for seedling vigour on regular basis is likely to improve salinity tolerance and drought performance of the germplasm in the long run. However, the correlations between shoot dry weight under control and salt stress were mostly moderate (Table 3) indicating that only selecting for seedling vigour would not improve salt tolerance of the germplasm by a great extent. It would be desirable to use other approaches to improve the salt tolerance in a breeding program.

In present study, we manually scored the salt stress of field pea seedlings from salt stress treatment based on a 1–10 scale. In our study, visual scores consistently had strong negative correlations with STI across the four experiments (Table 3) suggesting that the visual scoring effectively captured the genotypic differences in salinity tolerance. This was further supported by positive correlations of visual scores with shoot Na^+^ but negative correlations with shoot K^+^ and shoot K^+^/Na^+^ (Table 4). Large-scale screening for salt tolerance is costly when it needs both the control and salt stress treatments. Our study showed that visual score and shoot dry weight from salt stressed treatment can be effectively used to assess the salinity tolerance. This approach does not require control treatment and hence would save labour and other resources.

## 5. Conclusions

We conducted four pot experiments in a semi-controlled environment to assess the contribution of seedling vigour in salinity tolerance of field pea seedlings. Shoot dry weight under salt stress was consistently associated with improved tolerance to salt stress indicated by higher salt tolerance index and lower visual scores of salt stress. Seedling vigour (shoot dry weight under control) did not show consistent correlations with salt tolerance index. However, it had consistent and moderate correlations with shoot dry weight under salt stress, lower tissue damage symptoms and lower Na^+^ concentrations in shoot, indicating that seedling vigour indirectly contributed to salt tolerance of field pea seedlings. Salt tolerant genotypes had lower shoot Na^+^ concentration but higher root Na^+^ concentration than the susceptible genotypes, indicating that Na^+^ exclusion was the underlying process of salt stress tolerance in the field pea germplasm. As the visual scores were associated with salt tolerance and shoot dry weight under salt stress, visual scores and shoot dry weight under salt stress could be used to assess the salt stress tolerance of field pea seedlings. This would require only one treatment (i.e. salt treatment) in the experiments and would save resources.

## Figures and Tables

**Table 1:** Visual scoring of salinity stress symptoms.

Salinity Symptom Score (1–10)	Description
1	Plant healthy green, no obvious salinity symptoms.
2	Beginning to yellow, not very many symptoms.
3	Some chlorosis bottom half of plant, no necrosis, overall yellowing.
4	Necrosis beginning on bottom half of plant.
5	Chlorosis and necrosis bottom half of plant, yellowing overall (50% affected).
6	Chlorosis becoming more severe on upper part of plant, not necrotic on upper plant.
7	Chlorosis and necrosis more than half of plant.
8	More necrosis than 7, but still some green leaves.
9	Stem and very youngest leaves green, rest dead (all leaves may be dead).
9.5	Only top of stem (or small part stem) and very youngest leaves still green, rest dead (all leaves may be dead).
10	Plant dead.

**Table 2:** REML variance components analysis for field pea above ground biomass with treatments as control/salt and subplots as genotypes.

Fixed Terms	2018		2019		2020		2021	
	Wald Statistic	*p* Value	Wald Statistic	*p* Value	Wald Statistic	*p* Value	Wald Statistic	*p* Value
Genotype (G)	749	<0.001	521	<0.001	770	<0.001	465.85	<0.001
Treatment (T)	8629	<0.001	143	<0.001	106	<0.001	455.44	<0.01
G × T	352	<0.001	1.56	0.96	293	<0.001	10.89	0.688
No of plants	234	<0.001	347	<0.001	81.2	<0.001	22.01	<0.001

Note: Fixed terms: constant, genotype, treatment, genotype x treatment, number of plants/pot. Random terms: block, block × treatment, block × treatment × genotype.

**Table 3:** Pearson’s correlation coefficients between traits measured in 2018–2020.

	Shoot DWctrl g/pot	Shoot DWstress g/pot	Salt Tolerance Index
2018 (n = 146)			
Shoot DWstress	0.46 **		
Salt tolerance index	−0.19 *	0.78 **	
Visual score	−0.36 **	−0.78 **	−0.61 **
2019 (n = 147)			
Shoot DWstress	0.59 **		
Salt tolerance index	−0.04	0.78 **	
Visual score	−0.27 **	−0.69 **	−0.66 **
2020 (n = 147)			
Shoot DWstress	0.47 **		
Salt tolerance index	−0.18 *	0.76 **	
Visual score	−0.18 *	−0.67 **	−0.61 **
2021 (n = 15)			
shoot DWstress	0.94 **		
Salt tolerance index	0.55 *	0.80 **	
Visual score	−0.82 **	−0.89 **	−0.76 **

DW = dry weight, ctrl = control, STI = salt tolerance index. Visual scores and elemental concentrations were recorded from salt stress treatments. *, ** indicate that the correlations were significant at 0.05 and 0.01 alpha levels, respectively.

**Table 4:** Pearson’s correlation coefficients of traits related to biomass and visual score with elemental concentrations and their ratios in 2021 (n = 15).

	Shoot DWctrl (g/pot)	Shoot DWstress (g/pot)	Salt Tolerance Index	Visual Score (1–10)
Shoot Na^+^	−0.53 *	−0.73 **	−0.84 **	0.79 **
Shoot K^+^	0.10	0.30	0.54 *	−0.45 *
Root Na^+^	0.39	0.57 *	0.71 **	−0.67 **
Root K^+^	0.72 **	0.64 **	0.32	−0.49 *
Shoot K^+^/Na^+^	0.41	0.61 **	0.74 **	−0.70 **
Shoot Ca^2+^/Na^+^	−0.08	0.14	0.45 *	−0.29
Root K^+^/Na^+^	0.19	−0.06	−0.46 *	0.30
Root Ca^2+^/Na^+^	−0.31	−0.49 *	−0.65 **	0.59 *

DW = dry weight, ctrl = control, STI = salt tolerance index. Visual scores and elemental concentrations were recorded from salt stress treatments. *, ** indicate that the correlations were significant at 0.05 and 0.01 alpha levels, respectively.

**Table 5:** Contributions of plant traits to the three main principal components (PCA). Values were obtained from a matrix of 11 traits and 15 field pea genotypes and are ranked in order of magnitude in PC1. The highest contribution per PC is indicated by an asterisk (*).

Plant Traits	PC1 (59.8%)	PC2 (20.0%)	PC3 (12.5%)
Shoot Na^+^ under salt stress	14.1 *	0.4	2.1
Salt tolerance index	12.3	0.6	0.1
Shoot K^+^/Na^+^ under salt stress	12	2.6	9.5
Visual scores of salt stress	11.6	6.5	0.2
Shoot DWstress	9.6	15.5	1.1
Root Na^+^ under salt stress	8.9	0.3	24.4
Root Ca^2+^/Na^+^ under salt stress	8.4	0	26.5 *
Shoot K^+^ under salt stress	6.5	9.4	17.7
Shoot Ca^2+^/Na^+^ under salt stress	6	21.2	2.5
Root K^+^/Na^+^ under salt stress	5.4	16.8	13.4
Shoot DWctrl	5.2	26.7 *	2.5

## Data Availability

All the data are available upon request.

## Supplementary Materials

The following supporting information can be downloaded at: https://www.mdpi.com/article/10.3390/plants13223140/s1, Table S1. Averages of the measurements recorded in 2018. SE = stardandard error of means. Table S2. Averages of the measurements reocorded in 2019. SE = stardandard error of means. Table S3. Averages of the measurements reocorded in 2020. SE = stardandard error of means. Table S4. Averages of the measurements recorded in 2021. SE = stardandard error of means. Table S5. Analysis of variance of root and shoot elemental concentrations and their ratios recorded in 2021.
